# A Potential Target for Diabetic Vascular Damage: High Glucose-Induced Monocyte Extracellular Vesicles Impair Endothelial Cells by Delivering miR-142-5p

**DOI:** 10.3389/fbioe.2022.913791

**Published:** 2022-05-09

**Authors:** Rui Zhang, Shuai Niu, Zhihua Rong, Fengshi Li, Leng Ni, Xiao Di, Changwei Liu

**Affiliations:** Department of Vascular Surgery, Peking Union Medical College Hospital, Chinese Academy of Medical Sciences and Peking Union Medical College, Beijing, China

**Keywords:** diabetes mellitus, extracellular vesicles, monocytes, endothelial damage, miR-142-5p

## Abstract

Endothelial dysfunction is a key accessory to diabetic cardiovascular complications, and the regulatory role of the extracellular vesicles (EVs) from the innate immune system is growing. We tested whether EVs derived from high glucose-induced monocytes could shuttle microRNAs and impair endothelial cells. EVs from high glucose- and basal glucose-treated THP-1 cells (^HG-^THP-1 EVs and ^BG-^THP-1 EVs) were isolated and identified. After coculture with THP-1 EVs, human umbilical vein endothelial cells (HUVECs) were tested by proliferation, migration, reactive oxygen species (ROS) detection assays, and western blot for Nrf2/NLRP3 signaling. MiR-142-5p was predicted by miRNAs databases and further verified by RT–qPCR and dual-luciferase reporter gene assays that inhibit Nrf2 expression. The regulation of miR-142-5p in HUVECs was further evaluated. A type 1 diabetes mellitus (T1DM) mouse model was developed for miR-142-5p inhibition. Aorta tissue was harvested for hematoxylin-eosin staining and immunohistochemistry of interleukin-1β (IL-1β). Compared to ^BG-^THP-1 EVs, ^HG-^THP-1 EVs significantly reduced migration and increased ROS production in HUVECs but did not affect proliferation. ^HG-^THP-1 EVs induced suppression of Nrf2 signaling and NLRP3 signaling activation. RT–qPCR results showed that ^HG-^THP-1 EVs overexpressed miR-142-5p in HUVECs. The transfection of miR-142-5p mimics into HUVECs exhibited consistent regulatory effects on ^HG-^THP-1 EVs, whereas miR-142-5p inhibitors demonstrated protective effects. The miR-142-5p antagomir significantly reduced the IL-1β level in T1DM aortas despite morphological changes. To conclude, miR-142-5p transferred by high glucose-induced monocyte EVs participates in diabetic endothelial damage. The inhibition of miR-142-5p could be a potential adjuvant to diabetic cardiovascular protection.

## Introduction

Diabetes mellitus (DM) is the leading cause of mortality due to increased risk for cardiovascular diseases. Dysfunction of vascular endothelial cells irritated by high glucose is a critical driver for diabetic vascular complications (Maruhashi and Higashi, 2021). High glucose induces extensive production of reactive oxygen species (ROS), which leads to DNA impairments and cell death of endothelial cells (Teodoro et al., 2018). Recent studies revealed a close relationship between the innate immune system and diabetic vascular complications, in which monocytes, once activated by high glucose, can still regulate the function of endothelial cells despite the glycemic control, leading to cardiovascular diseases (Hoogeveen et al., 2018; Thiem et al., 2019). The crosstalk between monocytes and endothelial cells may provide new preventive targets for diabetic endothelial damage.

Extracellular vesicles (EVs) are lipid-based nanoparticles which can be naturally secreted by cells (Sanwlani and Gangoda, 2021). EVs are able to mediate cellular regulation by shuttling nucleic acids, proteins, lipids between cells and organs (Jurgielewicz et al., 2021). MicroRNAs (miRNAs) are a group of highly conserved, endogenous small non-coding RNAs that regulate the expression of multiple genes (Bartel, 2004). MiRNAs can also be carried by EVs for cell-to-cell communication (Xu et al., 2021). Several studies have reported that monocyte-derived EVs can regulate the function of endothelial cells (Sáez et al., 2019; Hu et al., 2021). However, few studies have investigated the regulation of EV-mediated miRNAs during monocyte-to-endothelial cell communication under high glucose conditions. We predicted that monocyte-derived EVs participate in the regulation of endothelial cells by transporting miRNAs.

Nuclear factor erythroid 2-related factor 2 (Nrf2), encoded by the NFE2L2 gene, is a transcription factor that enhances the expression of antioxidant enzymes, including heme oxygenase-1 (HO-1), superoxide dismutase 2 (SOD2), and NAD(P)H quinone dehydrogenase 1 (NQO-1), and reduces the level of ROS production (Alonso-Piñeiro et al., 2021). Notably, the activation of Nrf2 signaling protects against hyperglycemia-related damage in endothelial cells, indicating the protective value of the Nrf2 activator (Sharma et al., 2017). The pyrin domain-containing protein 3 (NLRP3) inflammasome complex is a well-characterized trigger of canonical pathways in pyroptosis and induces the inflammation cascade *via* apoptosis-associated speck-like protein containing a C-terminal caspase recruitment domain (ASC) and caspase-1 activation. The subsequent maturation of proinflammatory cytokines and gasdermin D (GSDMD) cleavage lead to extensive inflammation and osmotic lysis of the cells (Song et al., 2022). The crosstalk between Nrf2 signaling and the NLRP3 pathway is essential in stress-induced damage to cells, and the related mechanism in diabetic endothelial injury remains to be investigated.

In this study, we found that EVs from high glucose-treated monocytes regulated Nrf2 signaling and NLRP3 signaling in endothelial cells and impaired endothelial function *via* miR-142-5p. We also demonstrated that inhibiting miR-142-5p could alleviate hyperglycemia-induced endothelial damage in a type 1 DM (T1DM) mice model.

## Materials and Methods

### Animals

The experimental protocol in this study was approved by the Animal Ethics Committee of the Peking Union Medical College (No. XHDW-2019-001). A total of 30 six-week-old male C57BL/6j mice were obtained from Beijing Vital River Laboratory Animal Technology Co., Ltd. and raised at the Laboratory Animal Center of Peking Union Medical College Hospital. All animals were maintained under 12 h:12 h light/dark cycle at a temperature of 22 ± 2°C with free access to adequate food and water. To assess the impacts of hyperglycemia and avoid the possible influence of dyslipidemia, we chose the T1DM mouse model for this study, which was developed by a single intraperitoneal injection of STZ (150 mg/kg; Sigma-Aldrich, Germany) after acclimatization. Diabetes was verified by blood glucose ≥16.7 mmol/L 1 week later. The animals were divided into four groups: 1) control group; 2) T1DM group; 3) T1DM mice with tail vein injection of antagomir-miR-145-5p (4 OD, 5′-AGU​AGU​GCU​UUC​UAC​UUU​AUG-3′, GenePharma, China) every 2 weeks; 4) T1DM mice with tail vein injection of antagomir-negative control (NC, 4 OD, 5′- CAG​UAC​UUU​UGU​GUA​GUA​CAA-3′, GenePharma, China) every 2 weeks. Blood glucose and bodyweight of mice were measured. After 14 weeks, all mice were anesthetized using intraperitoneal injection of pentobarbital (45 mg/kg), and aorta tissues were harvested.

### Cell Culture

The human monocyte cell line THP-1 cells, human umbilical vein endothelial cells (HUVECs), and 293 T cells were obtained from the Chinese Academy of Medical Sciences and Peking Union Medical College. THP-1 cells were cultured in RPMI 1640 medium (Gibco, Unites States) supplemented with 10% fetal bovine serum (FBS; Biological Industry, Israel). THP-1 Cells with a confluence of around 70% were cultured in RPMI 1640 with 10% exosome-depleted FBS (Systembio, Unites States) for EVs isolation. HUVECs and 293T cells were cultured in DMEM medium (Gibco, Unites States) containing 10% FBS and 1% penicillin/streptomycin (Gibco, Unites States). All cells were maintained at 37°C with 5% CO2. Differentiated cells were incubated under basal-glucose (BG, 5.5 mM) and high-glucose (HG, 33 mM) conditions produced by D-(+)-Glucose (Sigma-Aldrich, Germany).

### Cell Transfection

MiR-142-5p mimics/mimics-NC/inhibitors/inhibitors-NC (GenePharma, China) were transfected into HUVECs by using Lipofectamine 3000 (Invitrogen, Unites States). MiR-142-5p mimics (5′-CAU​AAA​GUA​GAA​AGC​ACU​ACU-3′) were used to up-regulate miR-142-5p level in HUVECs, whereas miR-142-5p inhibitors (5′-AGU​AGU​GCU​UUC​UAC​UUU​AUG-3′) were used to inhibit endogenous miR-142-5p. MiR-142-5p mimics-NC (5′-UUG​UAC​UAC​ACA​AAA​GUA​CUG-3′) and miR-142-5p inhibitors-NC (5′-CAG​UAC​UUU​UGU​GUA​GUA​CAA-3′) were served as negative control.

### THP-1 EVs Preparation and Identification

EVs were extracted from freshly prepared THP-1 cell-cultured supernatants by Umibio Exosome Isolation Kit (Umibio, China) (protocol in Supplementary Figure S1). Following quantification using the Micro BCA™ protein assay kit (Thermo Scientific, Unites States), EVs were stored at −80°C. THP-1 EVs were identified by transmission electron microscopy (TEM), Nanoparticle tracking analysis (NTA), and western blot for CD9 and CD63.

### MicroRNAs Prediction

Targetscan 7.2 (Friedman et al., 2009), miRDB (Liu and Wang, 2019), TarBase v.8 (Karagkouni et al., 2018) and PicTar (Krek et al., 2005) were used to identify the potential upstream miRNAs that targeted Nrf2 gene (NFE2L2). All predicted miRNAs with conserved sites in Targetscan were included. In miRDB, predicted miRNAs with targetscore >90 were selected. Top 15 predicted miRNAs in TarBase and PicTar were involved. Jvenn app were employed to overlap the collected miRNAs from the databases (Bardou et al., 2014).

### Protein Extraction and Western Blot

EVs and cells were lysed using RIPA lysis buffer (Beyotime, China), and supernatants were collected after centrifugation. Protein level was assessed by Micro BCATM protein assay kit (Thermo Scientific, Unites States). The extracted protein (15 μg/lane) were separated by 12.5% SDS-PAGE (Epizyme, China) and transferred to PVDF membranes that were probed overnight at 4°C in primary antibodies against CD9 (1:2000, #ab92726, Abcam, United States), CD63 (1:1000, #ab134045, Abcam, United States), Calnexin (1:1000, #A15631, Abclonal, China) Nrf2 (1:2000, #ab62352, Abcam, United States), HO-1 (1:1000, #ab13248, Abcam, United States), NQO-1 (1:1000, #ab80588, Abcam, United States), SOD2 (1:1000, #ab68155, Abcam, United States), NLRP3 (1:1000, #ab263899, Abcam, United States), Caspase-1 (1:1000, #ab207802, Abcam, United States), GSDMD-N (1:1000, #ab210070, Abcam, United States). β-actin (1:1000, #4970S, Cell Signaling Technology, United States) was used as endogenous control. Protein was detected by enhanced chemiluminescence and film exposure.

### Real-Time Quantitative PCR

QIAzol lysis reagent (Qiagen, Germany) was used for lysis of cells. Total RNA was isolated and purified by miRNeasy mini kit (Qiagen, Germany). 1 μg RNA was used in reverse transcription by using miRNA 1st Strand cDNA Synthesis Kit (by stem-loop) (Vazyme, China). Real-time qPCR was performed using miRNA Universal SYBR qPCR Master Mix (Vazyme, China). RT primer and PCR primer sets were designed and produced by GenePharma (China). The forward (F) and reverse (R) primers are presented as followed (5–3′): U6-F: CGC​TTC​GGC​AGC​ACA​TAT​AC; U6-R: TTC​ACG​AAT​TTG​CGT​GTC​ATC; hsa-miR-142-5p-F: TAT​GGT​TGT​TCT​CGT​CTC​TGT​GTC; hsa-miR-142-5p -R: AGC​TCG​CGC​ATA​AAG​TAG​AAA​G; hsa-miR-144-3p-F: CCT​CTC​ACC​CTC​CCT​ACA​GTA​TAG​AT; hsa-miR-144-3p-R: TAT​GGT​TGT​TCA​CGA​CTC​CTT​CAC; hsa-miR-153-3p-F: AGC​CGC​TTC​GCA​TAG​TCA​CA; hsa-miR-153-3p-R: AGA​GCA​GGG​TCC​GAG​GAT. The relative expression of target genes was calculated using the 2^−△△Ct^ method. Gene expressions the above miRNAs were normalized to U6.

### Immunofluorescent Assay

EVs were incubated with the PKH67 (Umibio, China). The labelled EVs were co-cultured with HUVECs for 12 h in a 24-well plate. After a trice wash of phosphate-buffered saline (PBS), Hoechst 33342 (Beyotime, China) was added to HUVECs for 10 min incubation and was followed by another wash of PBS. The immunofluorescence was examined by a fluorescence microscope (DMi8, Leica, Germany).

### Cell Proliferation Assay

The proliferation of cells was detected by CCK-8 (Cell Counting Kit-8) solution (Analysis Quiz, China). 2,000 cells/well of HUVECs were seeded in the 96-well plate (100 μl/well). 10 μl of CCK-8 solution was added for another 2-h incubation, and the absorbance at 450 nm of each well was measured with Synergy H1 microplate reader (Biotek, United States). Cell proliferation assay was performed in triplicates.

### ROS Activity

The ROS production was detected by 2′,7′-dichlorofluorescin diacetate (DCFH-DA) assay (Beyotime, China). HUVECs were incubated with DCFH-DA assay in no-FBS culture medium (1:1000) at 37°C for 30 min and then washed thrice with no-FBS culture medium. The cells were observed under the fluorescence microscope (DMi8, Leica, Germany). The integrated fluorescence intensity of ROS was evaluated.

### Migration Assay

Migration assay was conducted by Culture-Inserts 2 Well (Ibidi, Germany). In brief, the 2-well silicone inserts were placed at the bottom of a 24-well plate. 70 μl HUVECs suspension was added in both wells of the silicone inserts. After cell adhesion, the silicone inserts were removed, leaving a 500 μm cell-free gap. The monolayer of HUVECs was cultured in DMEM with 2% FBS. The region of migration was photographed at 0 and 48 h after intervention.

### Dual-Luciferase Reporter Gene Assay

Wild (WT) and mutant (MUT) sequences of the Nrf2 gene were designed according to the binding site of miR-142-5p in the 3′-UTR of Nrf2 with reference to Targetscan 7.2. The Vectors of Nrf2-WT and -MUT were conducted by GenePharma using pmirGLO Dual-Luciferase miRNA Target Expression Vector (Promega, United states). 293 T cells were transfected with the modified vectors and miR-142-5p mimics/mimics-NC using Lipofectamine 3000 (Invitrogen, United states). Following the instruction of luciferase detection kit (Beyotime, China), the luciferase activity in each group was then detected in TriStar^2^ S microplate reader (Berthold Technology, Germany). The ratio of Firefly/Rennila activity was calculated. Dual-Luciferase reporter gene assay was performed in triplicates.

### Hematoxylin-Eosin Staining and Immunohistochemistry

Tissue was preserved in 4% paraformaldehyde (Solarbio, China). The tissue was then treated with ethanol gradient, xylene, and paraffin-embedding. Paraffin-embedded slices were serially sectioned for hematoxylin-eosin staining (H&E) or immunohistochemistry (IHC). Dewaxing, rehydration, and antigen retrieval were conducted. Then, the tissue slice was blocked by 5% bovine serum albumin. After being washed by PBS, the slides were incubated with interleukin-1β (IL-1β) antibody (1:100, #A19635, Abclonal, China) at 4°C overnight. The slides were further incubated with horseradish peroxidase-conjugated anti-rabbit IgG (1:5000; Zhongshan Jinqiao Biotechnology, China) for 1 h and were followed by the treatment of diaminobenzidine. The expression level of IL-1β were quantified in terms of the percentage of area with positive staining.

### Statistical Analysis

All data are presented as the means value with standard deviation. GraphPad Prism version 7.0 and IBM SPSS Statistics version 25 were employed, and the differences were analyzed by the student’s *t*-test between the two groups. One-way analysis of variance (ANOVA) was used to compare multiple groups. A *p*-value less than 0.05 were considered statistically significant.

## Results

### Isolation and Identification of THP-1 EVs

**FIGURE 1:**
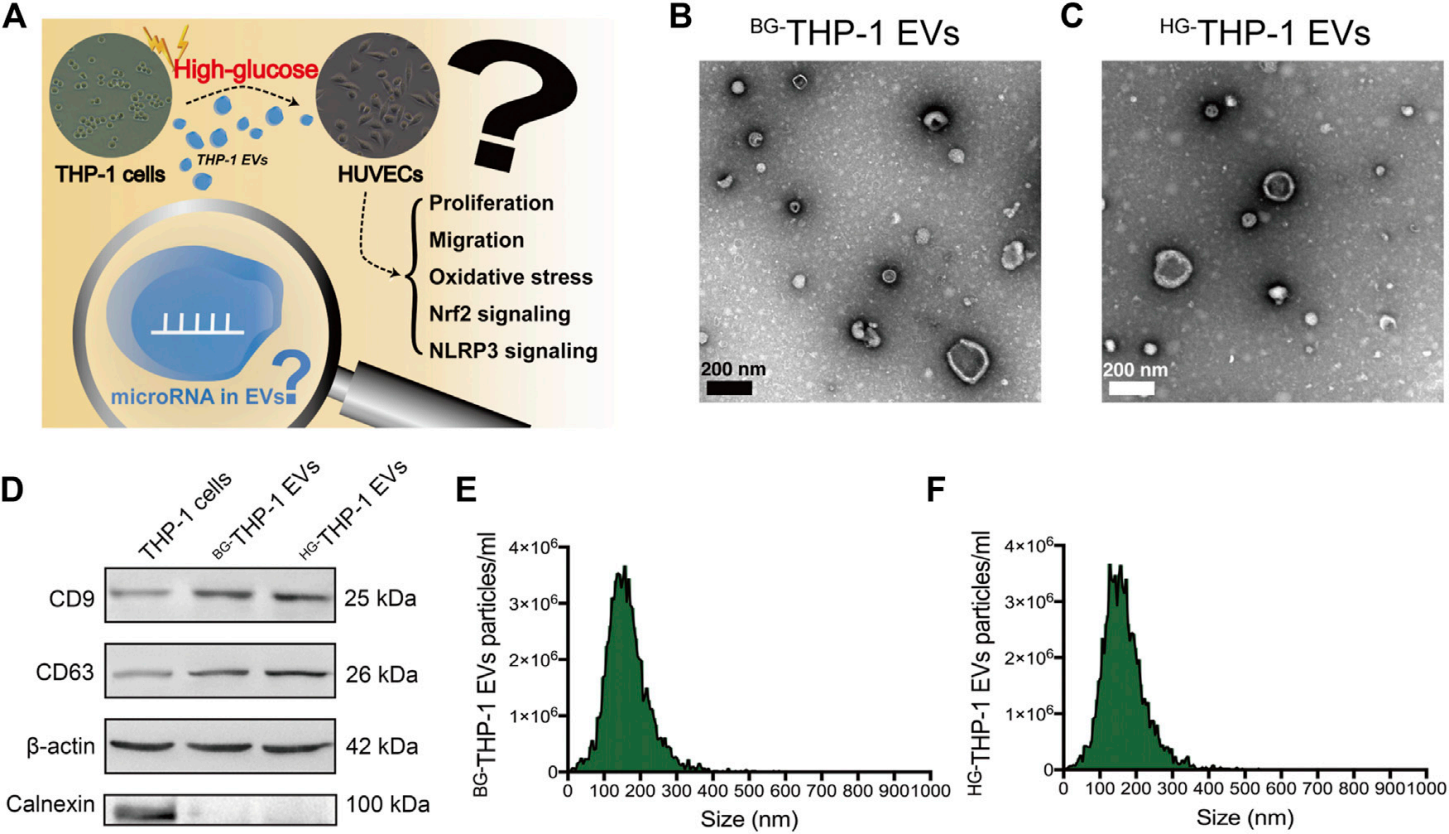
Isolation and identification of ^BG-^THP-1 EVs and ^HG-^THP-1 EVs **(A)** Schematic representation of study hypothesis and research question **(B,C)** Transmission electron microscopy for ^BG-^THP-1 EVs and ^HG-^THP-1 EVs, respectively. Scale bar = 200 nm **(D)** Western blot of CD9, CD63, Calnexin and β-actin in ^BG-^THP-1 EVs, ^HG-^THP-1 EVs, and THP-1 cells **(E,F)** Nanoparticle tracking analysis for ^BG-^THP-1 EVs and ^HG-^THP-1 EVs, respectively.

The scientific hypothesis is presented in Figure 1A. EVs derived from high glucose- and basal glucose-treated THP-1 cells (^HG-^THP-1 EVs and ^BG-^THP-1 EVs) were extracted from cultured supernatants. Under a transmission electron microscope, round and elliptical EVs with typical morphology were identified (Figures 1B,C). Western blot of EVs showed significantly increased expression of CD9 and CD63, whereas no expression of Calnexin was presented in EVs compared to THP-1 cells (Figure 1D). The NTA results suggested that the particle sizes range of ^BG-^THP-1 EVs and ^HG-^THP-1 EVs were 161.77 ± 60.16 nm and 162.66 ± 59.73 nm (Figures 1E,F, details were presented in Supplementary Table S1). These results verified the successful isolation of ^BG-^THP-1 EVs and ^HG-^THP-1 EVs.

### ^HG-^THP-1 EVs Reduces Migrating Potentials and Increases ROS Production in HUVECs

**FIGURE 2:**
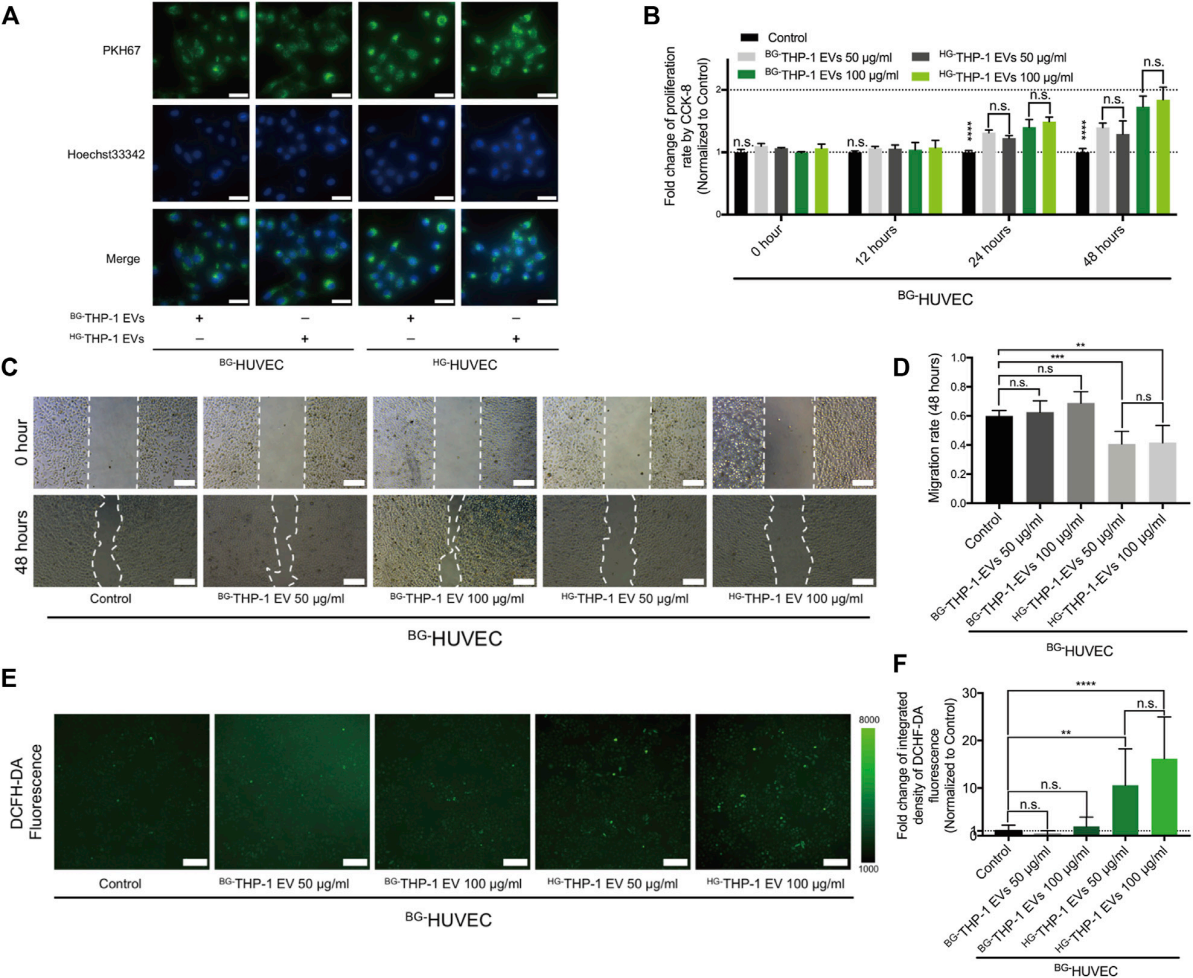
The regulatory effects of THP-1 EVs on HUVECs **(A)** The uptake of PKH67 (green fluorescent dye) -labelled THP-1 EVs (50 μg/ml) by HUVECs. Scale bar = 40 μm **(B)** The impact of THP-1 EVs on HUVECs proliferative ability **(C)** The impact of THP-1 EVs on HUVECs migration. Scale bar = 200 μm **(D)** Quantitative analysis of migration rate **(E)** The impact of THP-1 EVs on the production of ROS in HUVECs detected by DCFH-DA fluorescence. Scale bar = 200 μm **(F)** Quantitative analysis of DCFH-DA fluorescence. Data are presented as mean ± SD and analyzed using the ANOVA, n. s not significant, *p < 0.05, **p < 0.01, ***p < 0.001, ****p < 0.0001. *n* = 3.

^BG-^THP-1 EVs and ^HG-^THP-1 EVs were cocultured with HUVECs under basal glucose (^BG-^HUVEC) and high glucose (^HG-^HUVEC) conditions. Immunofluorescent staining showed the uptake of THP-1 EVs by HUVECs (Figure 2A). Both ^HG-^THP-1 EVs and ^BG-^THP-1 EVs enhanced the proliferation of HUVECs. However, there was no significant difference between the ^HG-^THP-1 EVs and ^BG-^THP-1 EVs groups (Figure 2B). Conversely, ^HG-^THP-1 EVs significantly reduced the migrative ability (Figures 2C,D) and increased ROS production of HUVECs (Figures 2E,F) compared to ^BG-^THP-1 EVs treatment. These results suggested that ^HG-^THP-1 EVs impaired the migration ability and increased oxidative stress in HUVECs.

### ^HG-^THP-1 EVs Regulates Nrf2 and NLRP3 Signaling in HUVECs

**FIGURE 3:**
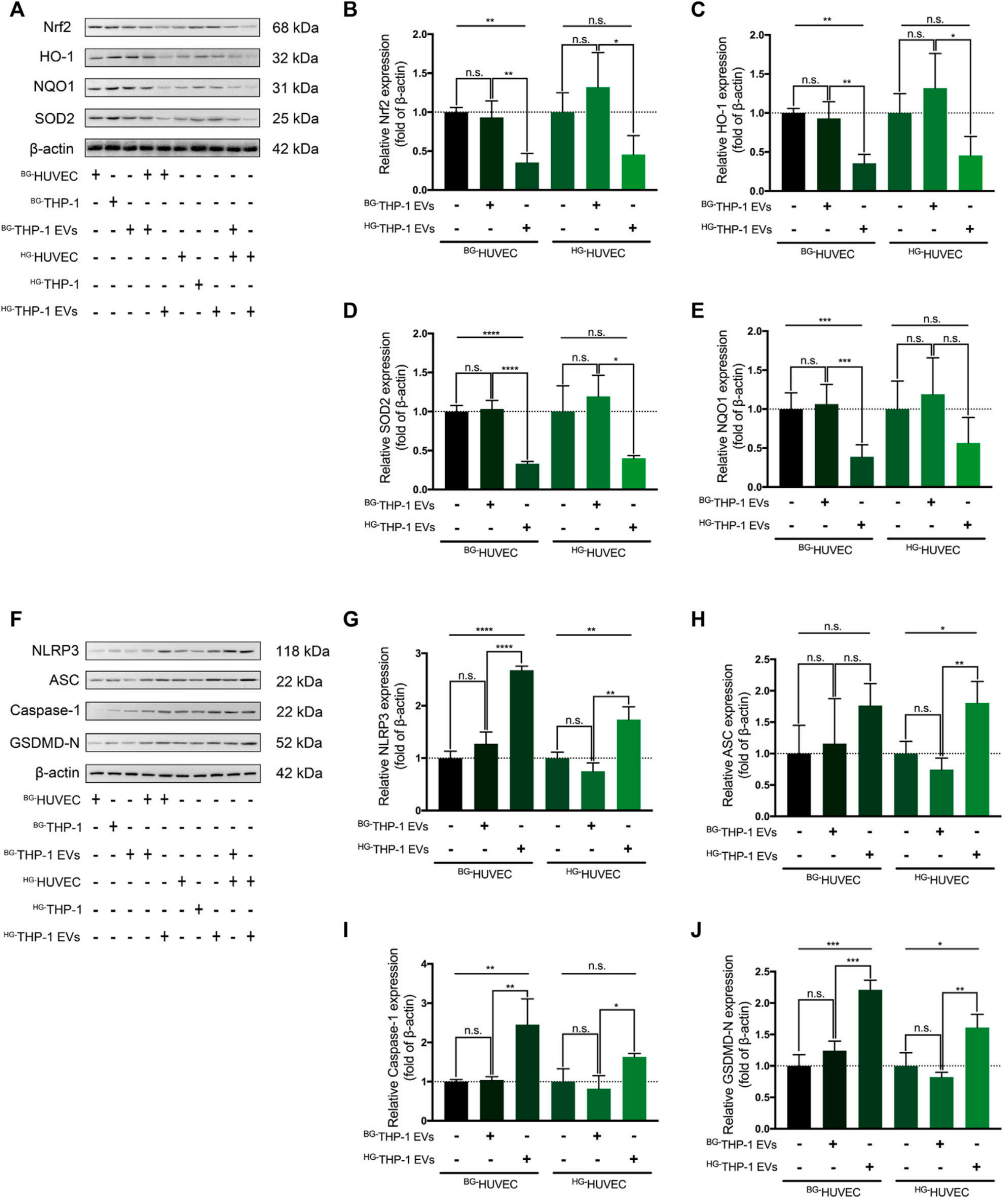
^HG-^THP-1 EVs regulate the Nrf2 and NLRP3 signaling pathway in HUVECs **(A–E)** THP-1 EVs regulating the expression of Nrf2, HO-1, SOD2, and NQO1 in HUVECs, in which ^HG-^THP-1 EVs significantly inhibited the level of the Nrf2 pathway signaling both in ^BG-^HUVEC and ^HG-^HUVEC **(F–J)** ^HG-^THP-1 EVs significantly increased the expression of NLRP3, ASC, Caspase-1, and GSDMD-N both in ^BG-^HUVEC and ^HG-^HUVEC. HUVECs, human umbilical vein endothelial cells. Data are presented as mean ± SD and analyzed using the ANOVA, n. s not significant, *p < 0.05, **p < 0.01, ***p < 0.001, ****p < 0.0001. *n* = 3.

In western blot analysis, ^BG-^THP-1 EVs did not elevate the expression of Nrf2, HO-1, SOD2, and NQO1 in HUVECs, whereas ^HG-^THP-1 EVs significantly decreased the level of Nrf2 signaling in HUVECs (Figures 3A–E). There was a noticeable inhibition of Nrf2 signaling when ^HG-^THP-1 EVs were added to ^HG-^HUVECs compared to ^BG-^THP-1 EVs. Moreover, there was no significant change in the expression level of NLRP3 signaling when ^BG-^THP-1 EVs were added to ^BG-^HUVECs or ^HG-^HUVECs. In comparison, ^HG-^THP-1 EVs elevated the levels of NLRP3, ASC, GSDMD-N, and Caspase-1 in both ^BG-^HUVECs and ^HG-^HUVECs (Figures 3F–J). The above results demonstrated that ^HG-^THP-1 EVs inhibited Nrf2 signaling and activated NLRP3 signaling in HUVECs.

### Nrf2-Targeting miR-142-5p Regulates Nrf2 and NLRP3 Signaling in ^HG-^HUVECs

**FIGURE 4:**
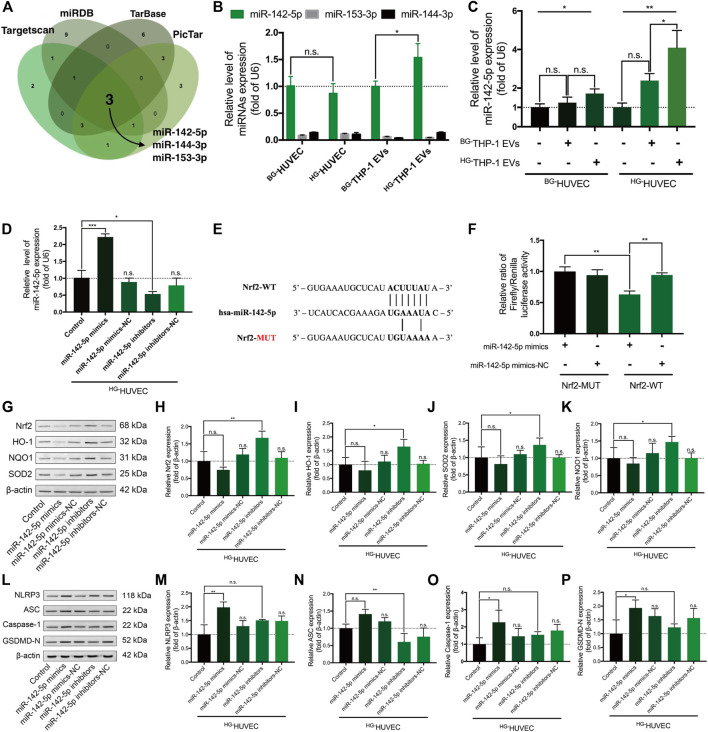
Nrf2-targeting miR-142-5p regulates the Nrf2 and NLRP3 signaling pathway in HUVECs **(A)** Predicted Nrf2-targeting microRNAs from four miRNAs databases **(B)** Relative expression of miR-142-5p, miR-153-3p, and miR-144-3p in HUVECs and in THP-1 EVs by RT-qPCR (by *t*-test) **(C)** Relative expression of miR-142-5p in HUVECs treated with THP-1 EVs **(D)** Relative expression of miR-142-5p in HUVECs with miR-142-5p mimics/mimics-NC/inhibitors/inhibitors-NC transfection **(E)** The binding sequence of miR-142-5p in the 3ʹ-UTR of Nrf2 transcript **(F)** Dual-luciferase assay verified the binding relationship between miR-142-5p and the 3ʹ-UTR of Nrf2 transcripts **(G–K)** MiR-142-5p regulating the expression of Nrf2, HO-1, SOD2, and NQO1 in ^HG-^HUVECs **(L–P)** MiR-142-5p regulating the expression of NLRP3, Caspase-1, ASC, and GSDMD-N in ^HG-^HUVECs. Data are presented as mean ± SD and analyzed using the ANOVA, n. s not significant, *p < 0.05, **p < 0.01, ***p < 0.001. *n* = 3.

TargetScan, Tarbase, miRDB, and PicTar suggested that miR-142-5p, miR-153-3p, and miR-144-3p may recognize and interact with the 3′-UTR of Nrf2 transcripts (Figure 4A, Supplementary Table S2). The RT–qPCR assay demonstrated that miR-142-5p had higher expression level in HUVECs and THP-1 EVs than miR-153-3p and miR-144-3p (*p* < 0.0001) (Figure 4B). Moreover, high-glucose environment did not alter the level of miR-142-5p in HUVECs, whereas the level of miR-142-5p significantly increased in ^HG-^THP-1 EVs than ^BG-^THP-1 EVs (Figure 4B). The expression of miR-142-5p was also elevated in HUVECs when treated with ^HG-^THP-1 EVs (Figure 4C). In addition, transfection of miR-142-5p mimics and inhibitors into HUVECs induced elevation and reduction in miR-142-5p levels, respectively (Figure 4D). A dual-luciferase reporter gene assay verified the target relationship between miR-142-5p and the 3′-UTR of Nrf2 transcript (Figures 4E,F).

In western blot analysis, miR-142-5p inhibitor significantly activated the Nrf2 signaling pathway. Moreover, there was a trend for miR-142-5p mimics reducing the level of Nrf2 signaling expression, although the change was not statistically significant (Figures 4G–K). MiR-142-5p mimics stimulated the up-regulation of NLRP3 signaling including NLRP3, Caspase-1, and GSDMD-N (Figures 4L–P). MiR-142-5p inhibitor prominently reduced the expression of ASC but not in NLRP3, Caspase-1, and GSDMD-N. Taken together, miR-142-5p regulates the Nrf2 signaling and the NLRP3 signaling pathway in ^HG-^HUVECs.

### MiR-142-5p Reduces Migrating Potentials and Increases ROS Production in ^HG-^HUVECs

**FIGURE 5:**
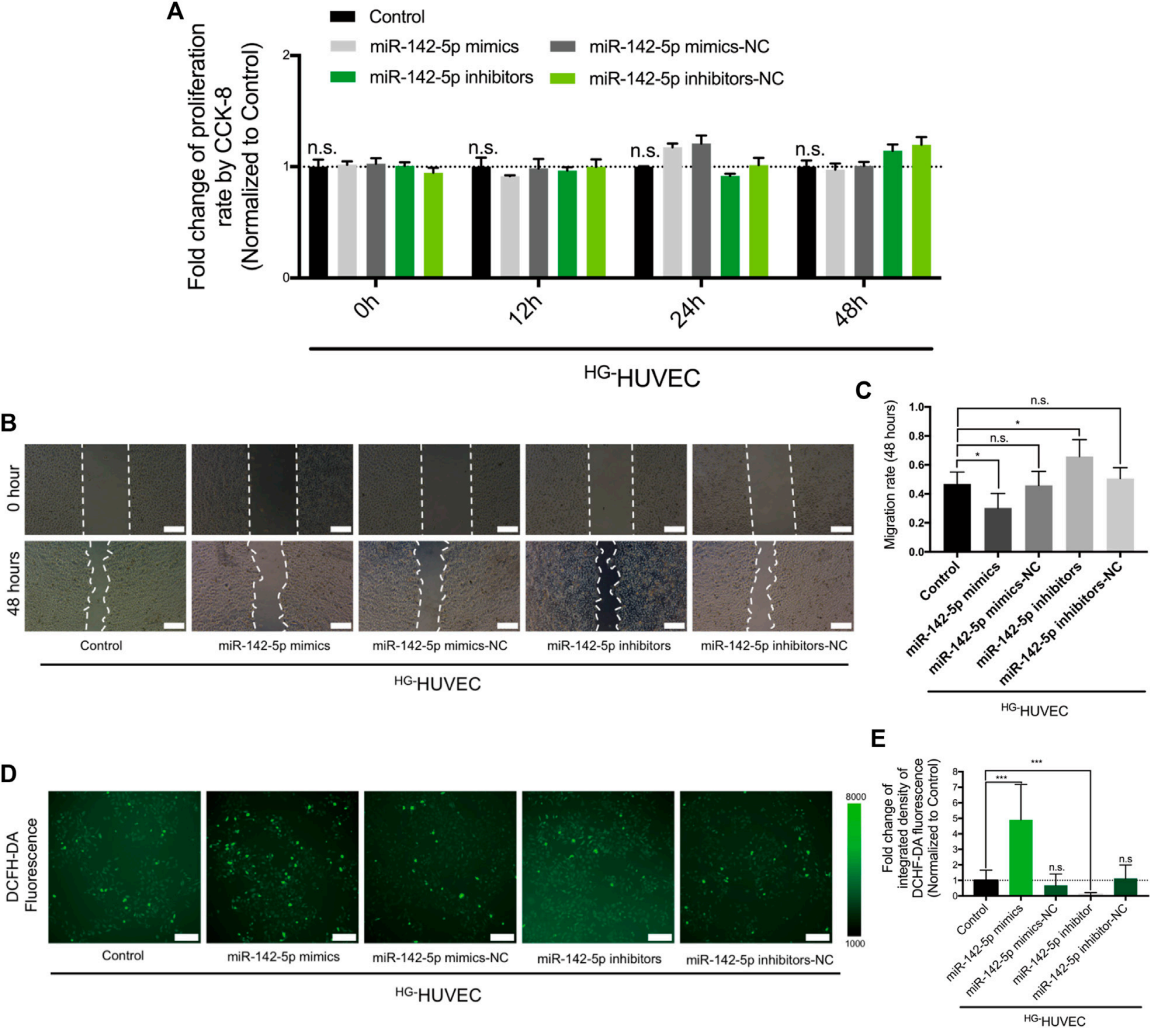
The regulatory effects of miR-142-5p on ^HG-^HUVECs **(A)** The impact of miR-142-5p on HUVECs proliferative ability **(B)** The impact of miR-142-5p on HUVECs migration. Scale bar = 200 μm **(C)** Quantitative analysis of migration rate **(D)** The impact of miR-142-5p on the production of ROS in HUVECs detected by DCFH-DA fluorescence. Scale bar = 200 μm **(E)** Quantitative analysis of DCFH-DA fluorescence. Data are presented as mean ± SD and analyzed using the ANOVA, n. s not significant, *p < 0.05, ***p < 0.001. *n* = 3.

We then tested how the differentially-expressed miR-142-5p between ^HG-^THP-1 EVs and ^BG-^THP-1 EVs affected the function of HUVECs. The results of CCK-8 showed that miR-142-5p did not impact the proliferative ability of HUVECs (Figure 5A). The transfection of miR-142-5p mimics limited the migratory abilities of HUVECs, and the migratory abilities of HUVECs were preserved in the miR-142-5p inhibitor group (Figures 5B,C). Furthermore, ROS production was significantly elevated in the miR-142-5p mimic groups, whereas miR-142-5p inhibitors reduced oxidative stress in HG-HUVECs (Figures 5D,E). The impacts of miR-142-5p on HUVECs were consistent with that of ^HG-^THP-1 EVs, indicating that ^HG-^THP-1 EVs regulated migration and ROS production *via* transferring Nrf2-targeting miR-142-5p to HUVECs.

### Inhibition of miR-142-5p Alleviates Inflammation in the Aortas of T1DM Mice

**FIGURE 6:**
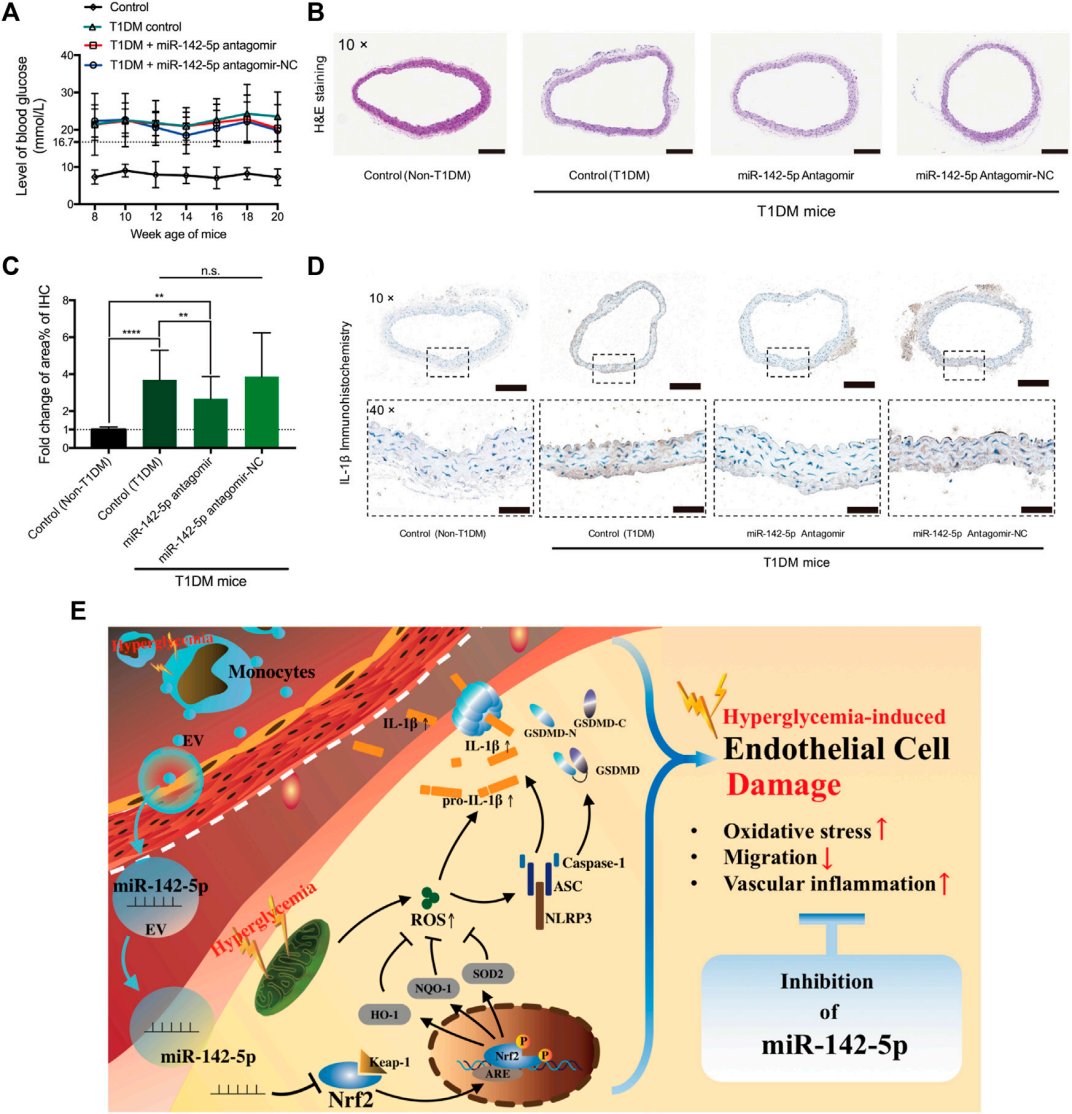
MiR-142-5p inhibition alleviates T1DM-induced vascular inflammation in the aorta **(A)** blood glucose level of mouse from four groups **(B)** Hematoxylin-eosin staining of aorta tissue harvested from mouse model at 20 weeks age (magnification, ×10). Scale bar = 200 μm **(C,D)** Relative fold change in positive staining area% of immunohistochemistry of IL-1β (magnification, ×10 & ×40). Scale bar = 200 & 50 μm) **(E)** Schematic diagram for the study findings. Data are presented as mean ± SD and analyzed using the ANOVA, n. s not significant, **p < 0.01, ****p < 0.0001. *n* = 5.

T1DM mice model was developed (Figure 6A). The results of H&E staining did not show apparent morphological changes among the groups (Figure 6B). IHC staining results revealed a significantly increased IL-1β level in T1DM mice compared to non-T1DM mice (Figures 6C,D). MiR-142-5p antagomir significantly reduced the level of IL-1β compared to the T1DM mice group. Despite the miR-142-5p antagomir treatment, the expression of IL-1β was still notably higher than the control group (Figures 6C,D). These results suggested that inhibition of miR-142-5p attenuates vascular inflammation induced by T1DM.

## Discussion

The pivotal role of EVs was identified in a variety of diseases and pathogenetic mechanisms (Grieco et al., 2021). Considering the lipid envelops structure and cargo-delivery ability, EVs were involved in cell-to-cell communication of different cell types (Sanwlani and Gangoda, 2021). In the present study, we observed that ^HG-^THP-1 EVs impaired migration ability and increased oxidative stress in HUVECs. Moreover, ^HG-^THP-1 EVs down-regulated the level of Nrf2 signaling and aggravated NLRP3 signaling in HUVECs. These results expand the findings revealed by Sáez et al. (2019) that high glucose-induced EVs from another monocyte cell line stimulated the proinflammatory state of endothelial cells. Oxidized low-density lipoprotein and lipopolysaccharide could also stimulate monocyte EVs, which conferred inflammation in endothelial cells (Puhm et al., 2019; Hu et al., 2021). These findings support the emerging role innate immune system in regulating the pathogenesis of endothelial dysfunction triggered by oxidative stress.

We and others previously found that by inhibiting NLRP3 signaling *via* Nrf2 signaling activation, endothelial cells were protected against stress from cigarette smoke extract (Hu et al., 2018; Zhao Z. et al., 2021; Jin et al., 2021). A series of studies found that stressors, including smoking and high glucose, stimulated inflammation in vascular cells *via* Nrf2 downregulation (Miao et al., 2013; Wang et al., 2021). The activation of Nrf2 ameliorated vascular inflammation and the progression of atherosclerosis in the diabetic setting (Hur et al., 2010). Previously, we demonstrated that the activation of the Nrf2 signaling pathway by melatonin protected against diabetes-related oxidative stress in vascular smooth muscle cells (Wang et al., 2021). The role of Nrf2/NLRP3 regulation has also been identified in a model of diabetic retinopathy and nephropathy (Liu et al., 2018; Abd El-Khalik et al., 2021). In this study, we first reported that ^HG-^THP-1 EVs modulated Nrf2 signaling and NLRP3 signaling, suggesting the proinflammatory role of ^HG-^THP-1 EVs compared to ^HG-^THP-1 EVs.

Cumulative evidence suggests that miRNAs derived from EVs participate in the pathogenesis of cardiovascular complications (Zhao S. et al., 2021). In this study, we identified that miR-142-5p was overexpressed in ^HG-^THP-1 EVs. The regulatory relationship between miR-142-5p and Nrf2 expression was verified by dual-luciferase reporter gene assay and western blot analysis. MiR-142-5p inhibitors significantly elevated Nrf2, HO-1, SOD2, and NQO1 expression, whereas the transfection of miR-142-5p mimics showed a trend of reduced expression of the Nrf2 signaling pathway. The comparatively lower expression level of Nrf2 in ^HG-^HUVECs may lead to statistically insignificance. MiR-142-5p mimics activated the NLRP3 signaling pathway, indicating the proinflammatory role of miR-142-5p. However, the inhibition of miR-142-5p only decrease the level of ASC expression in the NLRP3 signaling pathway. This intriguing finding may be related to other signaling pathway that miR-142-5p inhibitors were involved, which require further exploration.

Notably, the transfection of miR-142-5p mimics into HUVECs exhibited consistent regulatory effects on ^HG-^THP-1 EVs, which indicated that miR-142-5p played a key role in the endothelial dysfunction activated by ^HG-^THP-1 EVs. The upregulation of miR-142-5p was associated with cell dysfunction and death (Lou et al., 2017). Moreover, increased expression of miR-142-5p was found in plaques in an atherosclerotic animal model (Xu et al., 2015). Circulating miR-142-5p was positively associated with aggravation of vascular in-stent restenosis, which is a complication significantly attributed to endothelial cell inflammation and dysfunction (Pan et al., 2021). One publication demonstrated that the inhibition of miR-142-5p reduced high-glucose induced inflammation in human retinal endothelial cells (Liu et al., 2020, 1). In the *in-vivo* study, we further explored the potential protective role of miR-142-5p inhibition in T1DM mice model. The findings revealed that miR-142-5p antagomir effectively reduced IL-1β expression in the mice aorta, but still failed to completely reverse the T1DM-induced vascular inflammation. This indicated that miR-142-5p inhibition is a potential adjuvant to glycemic control in terms of diabetic vascular damage.

There are several limitations of this study. In DM patients, endothelial cells are mediated by a number of factors including hyperglycemia, dyslipidemia, and fluctuating blood pressure. Thus, both *in-vitro* and *in-vivo* experiments do not fully reflect human settings. THP-1 EVs may also contain other pro-inflammatory contents. Despite the protective effects of miR-142-5p inhibition, it is unknown whether solely suppressing miR-142-5p in monocyte EVs could reverse DM-associated vascular damage. Detailed interactive mechanisms between monocytes and endothelial cells against the background of diabetes require future investigation. Still, our findings showed that ^HG-^THP-1 EVs induce endothelial dysfunction. MiR-142-5p is involved in this mechanism and elicits the potential in reducing high glucose-induced endothelial damage. This study provides a novel target against diabetic endothelial damage in addition to glycemic control.

## Conclusion

This study demonstrated that high glucose-induced monocyte EVs transferring miR-142-5p participate in diabetic endothelial damage, along with the regulation of Nrf2 and NLRP3 signaling. Vascular inflammation was reduced by inhibiting miR-142-5p in a T1DM mouse model. The present study provides novel insights into the pathogenesis of diabetic endothelial damage and suggests that the inhibition of miR-142-5p could be a potential adjuvant to diabetic cardiovascular protection.

## Data Availability

The original contributions presented in the study are included in the article/Supplementary Material, further inquiries can be directed to the corresponding author.
