# Supplementary material for: A Potential Target for Diabetic Vascular Damage: High Glucose-Induced Monocyte Extracellular Vesicles Impair Endothelial Cells by Delivering miR-142-5p
Source: Front Bioeng Biotechnol. 2022 May 9;10:913791. doi: 10.3389/fbioe.2022.913791 (PMC9124888; doi:10.3389/fbioe.2022.913791)
Supplement: Supplementary file 1 [file DataSheet1.DOCX]

Supplementary Material

# Supplementary Figures and Tables

|  |  | BG-THP-1 EVs | | HG-THP-1 EVs | |
| --- | --- | --- | --- | --- | --- |
|  |  | Statistic | Std. Error | Statistic | Std. Error |
| Mean |  | 164.8329 | 0.00656 | 165.782 | 0.00651 |
| 5% Trimmed Mean | | 161.7678 |  | 162.6568 |  |
| Median |  | 157.5 |  | 157.5 |  |
| Variance |  | 3618.831 |  | 3567.525 |  |
| Std. Deviation | | 60.15672 |  | 59.72876 |  |
| Minimum |  | 7.5 |  | 7.5 |  |
| Maximum |  | 582.5 |  | 537.5 |  |
| Range |  | 575 |  | 530 |  |
| Interquartile Range | | 65 |  | 70 |  |
| Skewness |  | 1.191 | 0 | 1.068 | 0 |
| Kurtosis |  | 3.936 | 0.001 | 2.71 | 0.001 |

**Supplementary Table 1.** Detailed statistical analysis of NTA.

| Targetscan | miRDB | Tarbase | PicTar |
| --- | --- | --- | --- |
| hsa-miR-708-5p | **hsa-miR-144-3p** | **hsa-miR-142-5p** | hsa-miR-23b-3p |
| hsa-miR-27a-3p | hsa-miR-3680-3p | hsa-miR-27a-3p | hsa-miR-130a-5p |
| hsa-miR-27b-3p | hsa-miR-6854-5p | **hsa-miR-153-3p** | **hsa-miR-144-3p** |
| hsa-miR-216a-3p | hsa-miR-132-3p | hsa-miR-101-3p | **hsa-miR-144-3p** |
| hsa-miR-3681-3p | hsa-miR-212-3p | hsa-miR-27b-3p | **hsa-miR-142-5p** |
| hsa-miR-128-3p | hsa-miR-582-5p | **hsa-miR-144-3p** | hsa-miR-128-3p |
| **hsa-miR-142-5p** | hsa-miR-340-5p | hsa-miR-20a-5p | hsa-miR-216a-3p |
| hsa-miR-5590-3p | hsa-miR-450b-5p | hsa-miR-340-5p | hsa-miR-5590-3p |
| hsa-miR-340-5p | **hsa-miR-153-3p** | hsa-miR-27b-5p | hsa-miR-23c |
| **hsa-miR-153-3p** | hsa-miR-7-1-3p | hsa-miR-23b-3p | hsa-miR-522-3p |
| **hsa-miR-144-3p** | hsa-miR-5590-3p | hsa-miR-182-5p | hsa-miR-101-3p |
| hsa-miR-140-5p | hsa-miR-500a-5p | hsa-miR-23a-3p | hsa-miR-27b-3p |
| **hsa-miR-144-3p** | hsa-miR-140-5p | hsa-miR-32-3p | hsa-miR-23a-3p |
|  | **hsa-miR-142-5p** | hsa-miR-199a-3p | hsa-miR-27a-3p |
|  | hsa-miR-7-2-3p | hsa-miR-128-3p | **hsa-miR-153-3p** |

**Supplementary Table 2.** Predicted miRNAs from Targetscan, miRDB, Tarbase, PicTar databases (Accessed date: 2021.5).

## Supplementary Figures


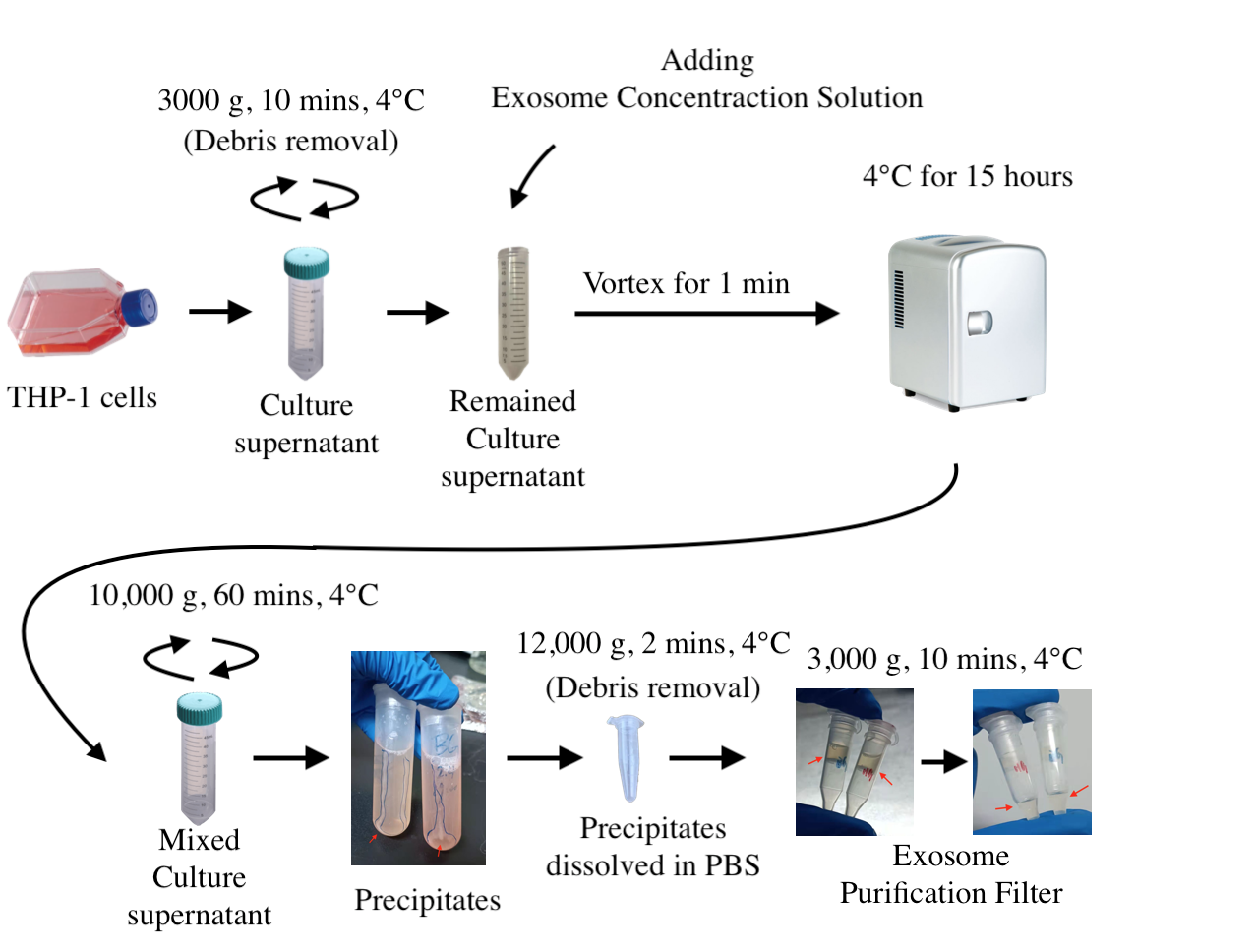


**Supplementary Figure 1.** Protocol of EVs isolation from THP-1 cells cultured supernatants according to the manufacturer’s instruction (#UR52121, Umibio, China).
